# Supplementary material for: Biophysical characterization of the CXC chemokine receptor 2 ligands
Source: PLoS One. 2024 Apr 16;19(4):e0298418. doi: 10.1371/journal.pone.0298418 (PMC11020491; doi:10.1371/journal.pone.0298418)
Supplement: S1 File — (ZIP) [file pone.0298418.s001.zip › SI_Final.docx]

**Biophysical Characterization of the CXC Chemokine Receptor 2 Ligands**

**Short title: Chemokine CXC-ligands**

Patrick Martin^1*^, Emily A. Kurth^1*^, David Budean^1*^, Nathalie Momplaisir^2^, Elaine Qu^2^Jennifer M. Simien^1^, Grace E. Orellana^1^, Chad Brautigam^3^, Alan V. Smrcka^2^, and Ellinor Haglund^1‡^

1. Department of Chemistry, University of Hawaii at Manoa, Honolulu, HI, 96822, USA

2. Department of Pharmacology, University of Michigan Medical School, Ann Arbor, MI, 48109, USA

3. Department of Biophysics and the Department of Microbiology, University of Texas Southwestern Medical Center, Dallas, TX, 75390, USA

*The authors contributed equally to this work.

^‡^To whom correspondence should be addressed: Ellinor Haglund (E-mail: ellinorh@hawaii.edu, Phone: +1(808) 956-5163).

**SUPPORTING INFORMATION**

The mass spectrometry data shows that our protein expression and purification protocol successfully purify CXC-ligands (**Figure S1**). The CD spectra combined with the activity data reveals that the CXC-ligands are folded into active CXCR2 agonist proteins (**Figure S1**). The identity of the CXC-ligands was verified with mass spectroscopy, where the measured mass-to-charge agrees with the calculated theoretical mass from the amino acids sequence of the mature proteins (**Figure S1**). Experimental conditions for dimeric CXC-ligands were determined using a combination of Analytical Ultracentrifugation (AUC) and equilibrium titrations at low, mid, and high protein concentrations (**Figure S2 and S3**).

To be able to monitor the folding reaction utilizing tryptophan (Trp) fluorescence, a Trp was introduced at position 58 and 62 for CXCL1 and CXCL5 respectively, designing CXCL1^I58W^ and CXCL5^F62W^ pseudo wild-type proteins (**Figures 3 and S4**). The equilibrium data shows that the introduction of a Trp residue affects the global stability for both proteins, shifting the ΔG = 3.68 ± 0.03 to 4.06 ± 0.04 kcal/mol for wild-type and CXCL1^I58W^ and ΔG = 5.57 ± 0.04 to 4.15 ± 0.08 kcal/mol for wild-type and CXCL5^F62W^ (**Figure S5 and Table S1 and S2**). The NMR experiments were conducted on a 600 MHz Bruker spectrometer at NMR Facility at Madison, Wisconsin (NMRFAM). The protein concentrations were between 60 to 100 μM in 10 mM phosphate buffer at pH 7.4 at 25 °C. The NMR HSQC displays minor shifts in the three-dimensional structure, indicative of no significant changes from the introduction of a Trp in CXCL1 and CXCL5. The activity data of the pseudo wild type-proteins show the same dose dependence for CXC-ligands and ERK phosphorylation. Overall, the designed pseudo wild-type proteins are good models for the CXC-ligands lacking a fluorescence probe.

The chevron plot displays a complex kinetic behavior with a fast and a slow phase (**Figure 3, and S4**). All observed rates and amplitudes for the CXC-ligands are plotted in **Figure S4**.

**SUPPORTING FIGURES**

**Figure S1.** Mass spectrometry of wild-type CXC-ligands. The data show that the purified protein has the mass-to-charge ratio corresponding to A) CXCL1 with a theoretical Mw: 7,865 kDa, B) CXCL5 with a theoretical Mw: 8,357 kDa, and C) CXCL8 with a theoretical Mw: 8,385 kDa. The bottom panel depicts the CD spectra for CXCL1, CXCL5, and CXCL8 respectively at 30 μM protein concentration.

**Figure S2.** Analytical Ultracentrifugation (AUC) of CXC-ligands. The graphs represent the CXC-ligands at low, mid, and high protein concentration using 10 mM phosphate buffer at pH 7.4 for A) CXCL1, B) CXCL5, and C) CXCL8.

**Figure S3.** Thermodynamic data of low, mid, and high protein concentrations. The CXC-ligands are depicted at 25 °C using 10 mM phosphate buffer at pH 7.4 for A) CXCL1, B) CXCL5, and C) CXCL8.

**Figure S4.** Kinetic phases observed for CXC-ligands. The overlay of fast and slow kinetics including the amplitude for the separate fits. The kinetics measured at low protein concentrations are plotted in black and blue, and high concentrations in red and gray for the fast and slow kinetics respectively. The unfolding kinetics was also measured in the presence of GdmCl, where cyan and green represent the fast and slow kinetics. The data for each CXC-ligand is plotted in A) CXCL1^I58W^, B) CXCL5^F62W^, and C) CXCL8.

**Figure S5.** The tryptophan variants of CXCL1 and CXCL5. A) CXCL1 wild-type versus CXCL1^I58W^. B) CXCL5 wild-type versus CXCL5^F62W^. The amino acid substitution introduces a fluorescent probe. Substituting isoleucine in CXCL1 and phenylalanine in CXCL5 at the N-terminus of the α-helix has a minor effect on the three-dimensional structure and the ΔG (Table S2). All proteins induce a comparable dose-dependent CXCR2-mediated ERK phosphorylation. The pseudo wild-type proteins cause a minor shift in the dose response, where CXCL1^I58W^ decreases the response and CXCL5^F62W^ increases the response, but maximal efficacy in mediating ERK phosphorylation relative to the wild-type proteins based on ANOVA analysis.

**SUPPORTING TABLES**

Table S1. Equilibrium titrations for CXC-ligands in 10 mM Phosphate buffer at pH 7.4 at 25 °C.

|  | MP  M | | m_D-N_  M^-1^ | ΔG  kcal/mol |
| --- | --- | --- | --- | --- |
| CXCL1 | 3.31 | 0.82 | | 3.68 ± 0.03 |
| CXCL1^I58W^ | 4.16 | 0.72 | | 4.06 ± 0.04 |
| CXCL5 | 3.32 | 1.23 | | 5.57 ± 0.04 |
| CXCL5^F62W^ | 4.58 | 0.67 | | 4.15 ± 0.08 |
| CXCL8 | 5.00 | 1.04 | | 7.07 ± 0.10 |

*Data fitted to a two-state equation.
