# Supplementary figures and images for: Biophysical characterization of the CXC chemokine receptor 2 ligands
Source: PLoS One. 2024 Apr 16;19(4):e0298418. doi: 10.1371/journal.pone.0298418 (PMC11020491; doi:10.1371/journal.pone.0298418)

A

CXCL1

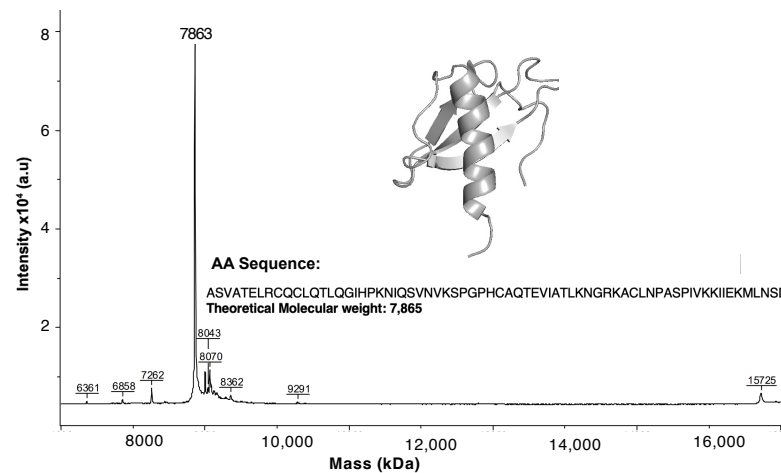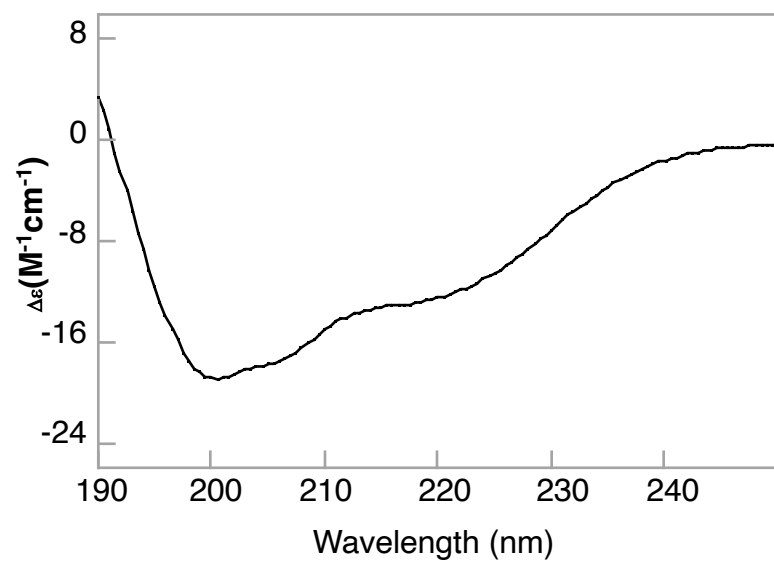

B

CXCL5

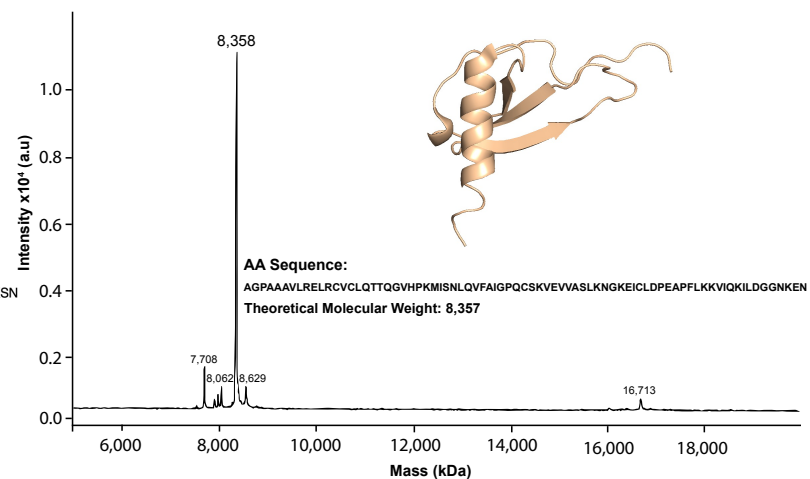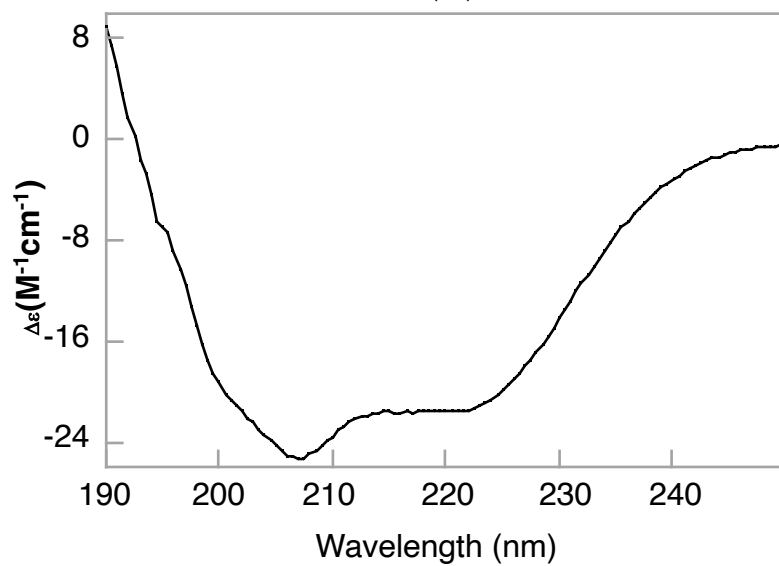

C

CXCL8

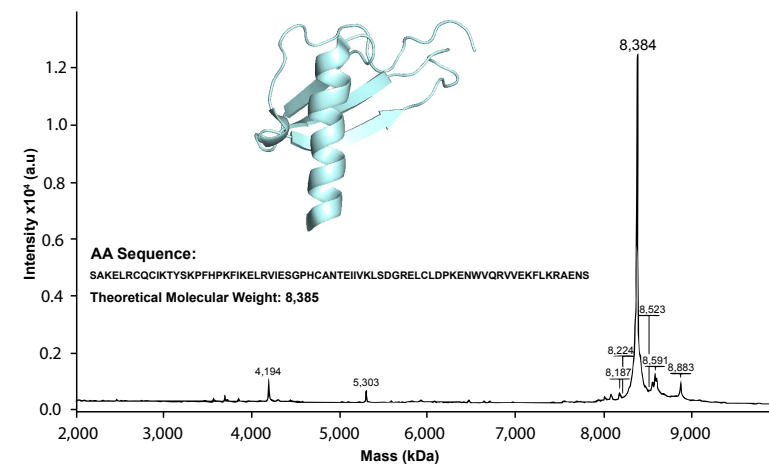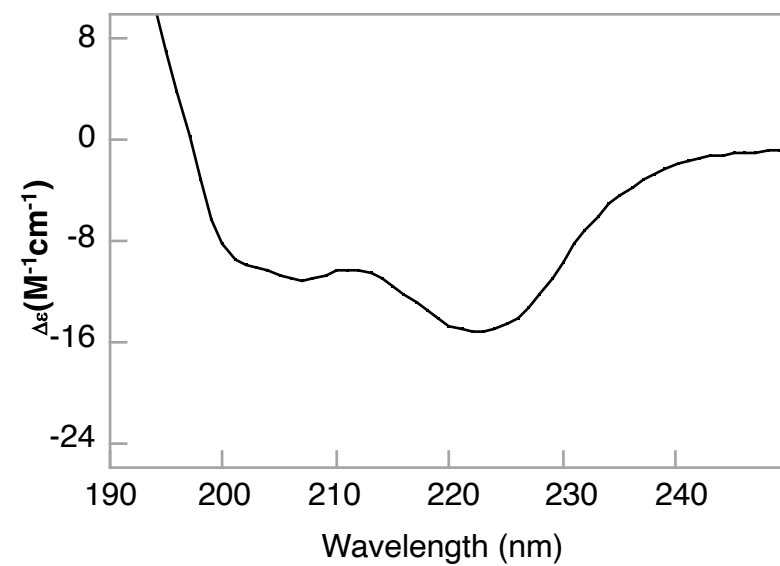

Supplement: S1 File — (ZIP) [file pone.0298418.s001.zip › Fig_S1.pdf]

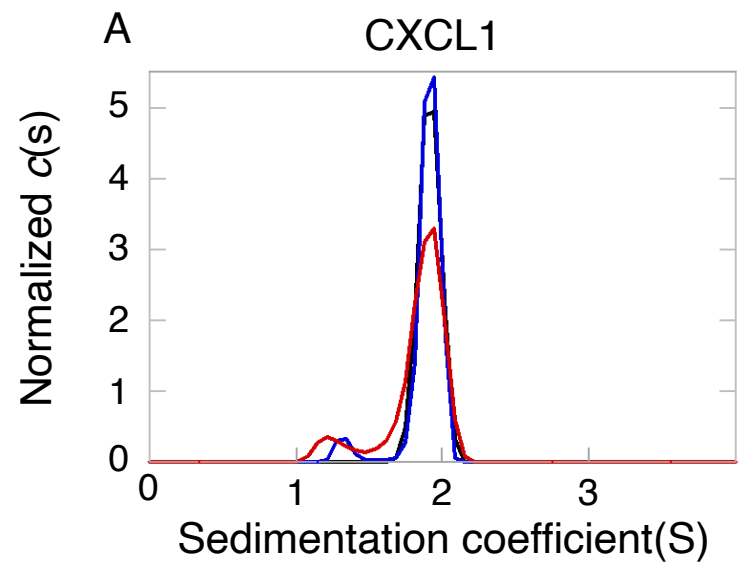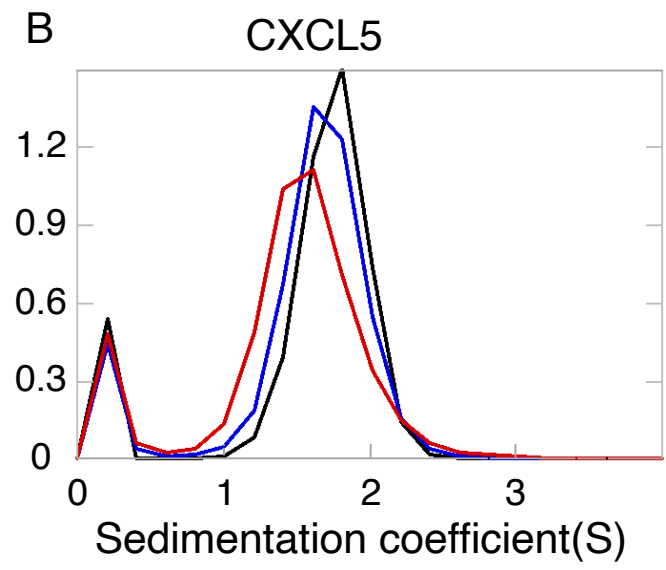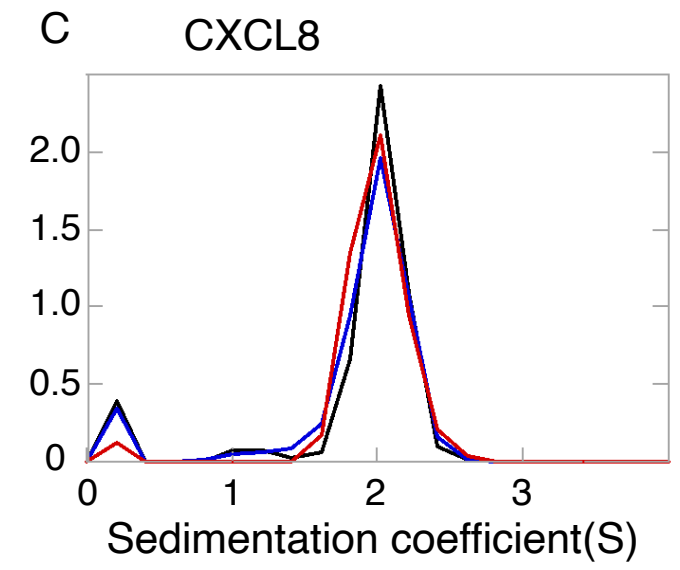

Supplement: S1 File — (ZIP) [file pone.0298418.s001.zip › Fig_S2.pdf]

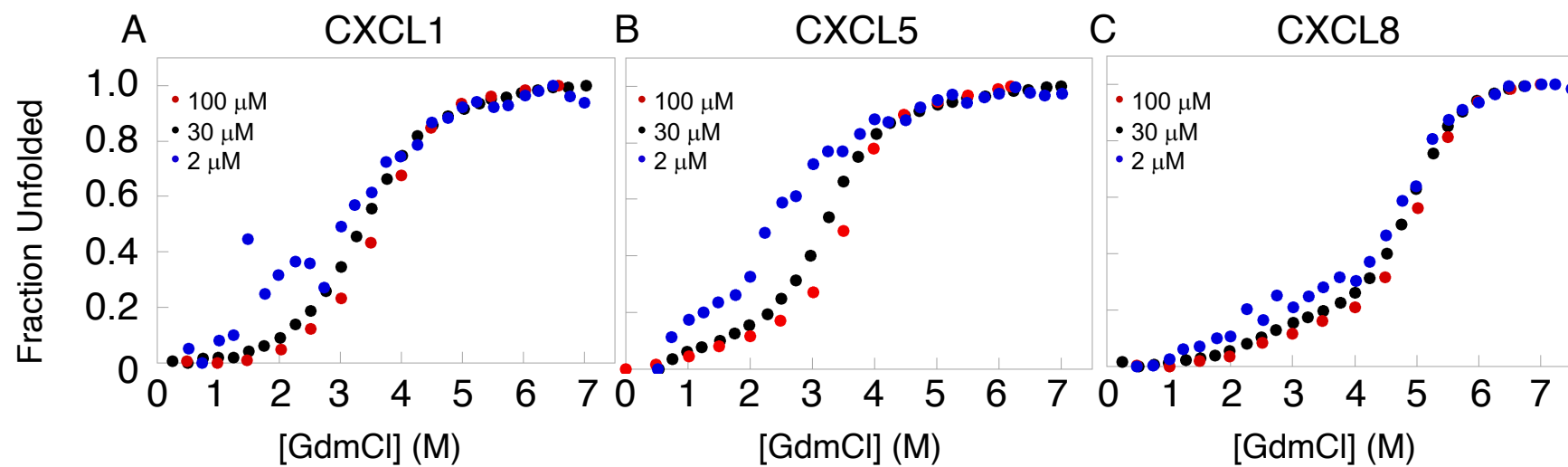

Supplement: S1 File — (ZIP) [file pone.0298418.s001.zip › Fig_S3.pdf]

A

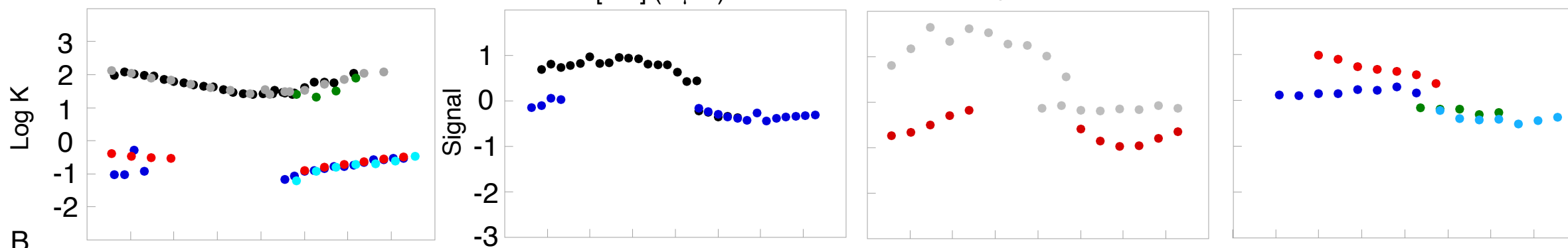

B

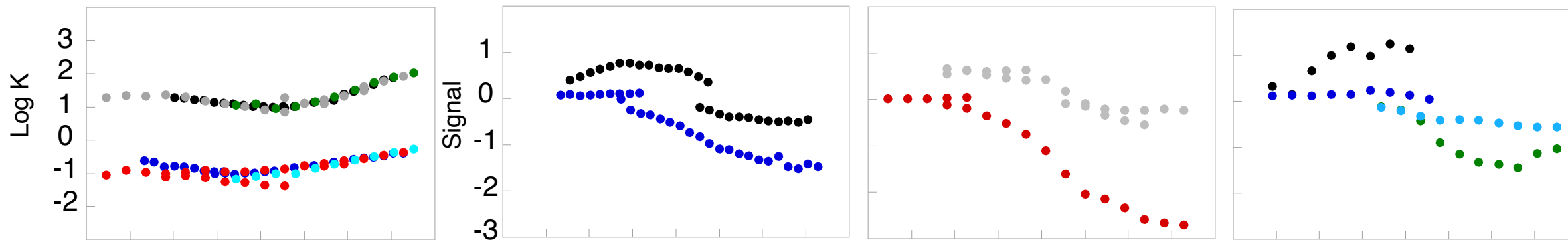

C

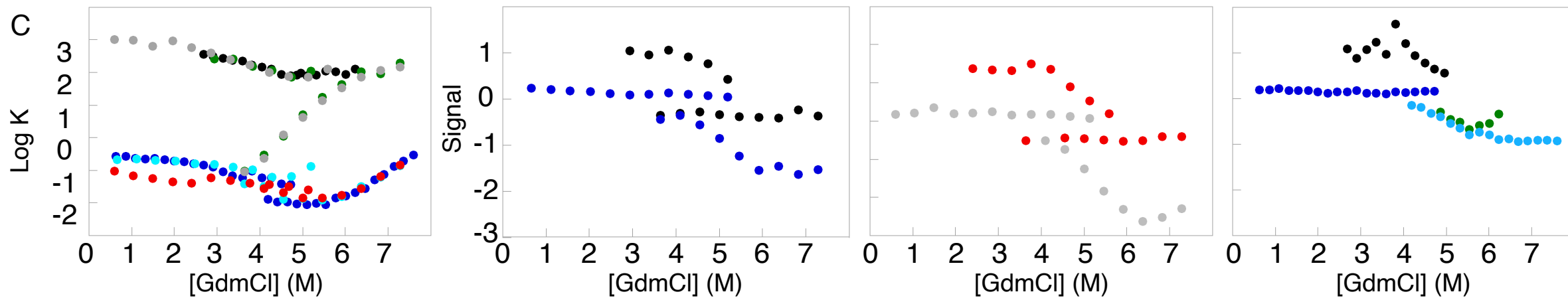

Supplement: S1 File — (ZIP) [file pone.0298418.s001.zip › Fig_S4.pdf]

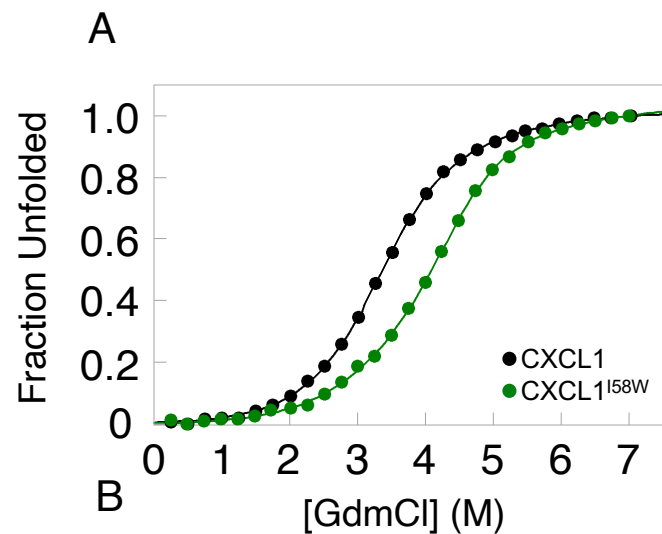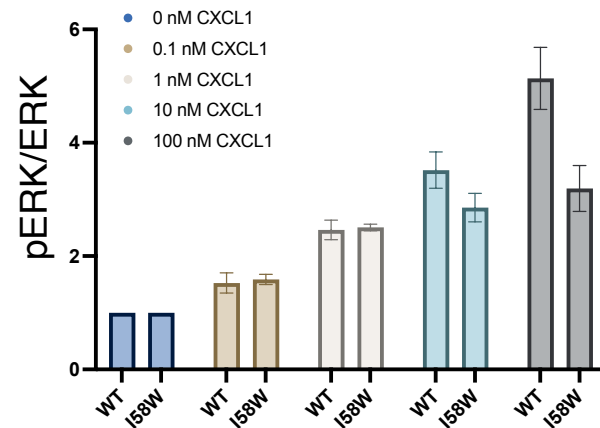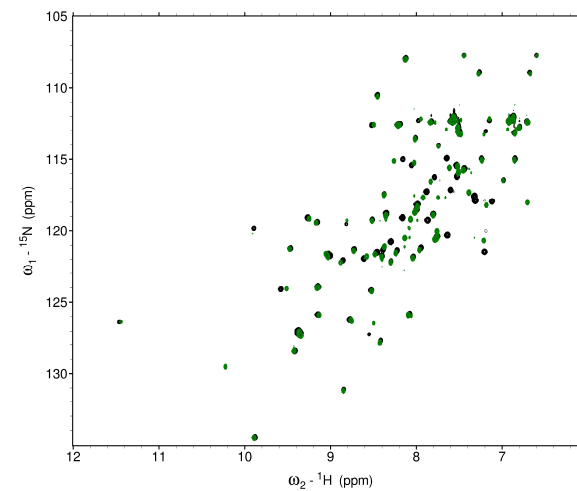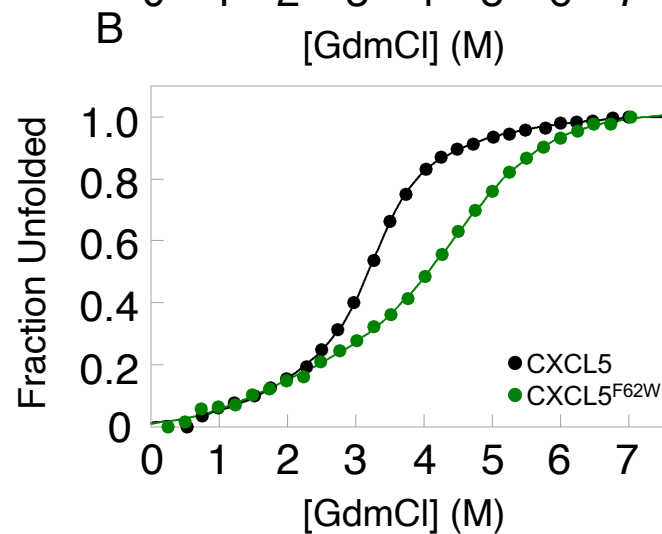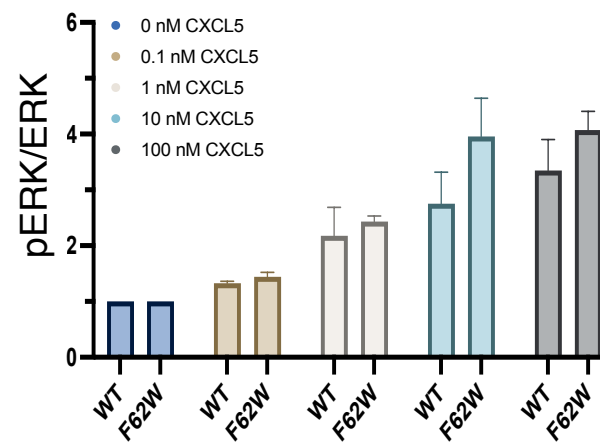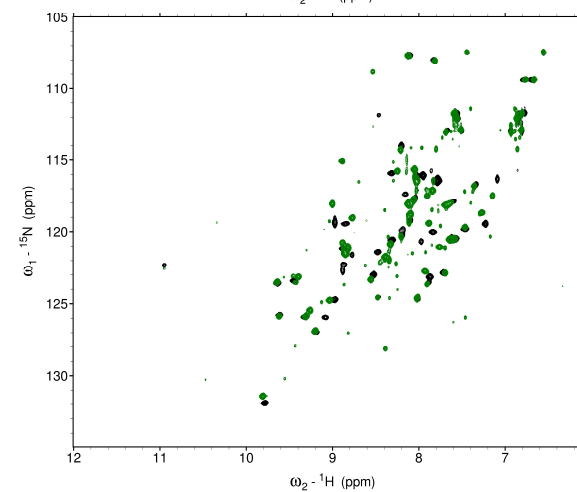

Supplement: S1 File — (ZIP) [file pone.0298418.s001.zip › Fig_S5.pdf]
